# RNA interference-based gene silencing of *phytoene synthase* impairs growth, carotenoids, and plastid phenotype in *Oncidium hybrid* orchid

**DOI:** 10.1186/2193-1801-3-478

**Published:** 2014-08-28

**Authors:** Jian-Xin Liu, Chung-Yi Chiou, Chin-Hui Shen, Peng-Jen Chen, Yao-Chung Liu, Chin-Der Jian, Xiao-Lan Shen, Fu-Quan Shen, Kai-Wun Yeh

**Affiliations:** Flower Research and Development Center, Zhejiang Academy of Agricultural Science, Hangzhou, 311202 Zhejiang China; Institute of Plant Biology, College of Life Science, National Taiwan University, Roosevelt Road, Taipei, 10617 Taiwan; Institute of Forestry Research, Council of Agriculture, Taipei, Taiwan; Ecological Materials Technology Department, Green Energy & Eco-technology System Center, ITRI South Campus, Industrial Technology Research Institute, Tainan, Taiwan; Institute of Bioinformatics and Structural Biology & Department of Life Science, National Tsing Hua University, 101, Sec. 2, Kuang-Fu Road, Hsinchu, 30013 Taiwan

**Keywords:** Carotenoid, *Oncidium* orchid, Phytoene synthase, Plastid, RNA interference

## Abstract

Phytoene synthase (PSY) is the first rate-limiting regulatory enzyme in the carotenoid biosynthesis pathway. In order to modify the floral color pattern by reducing carotenoid contents, a *phytoene synthase*-RNAi construct was delivered into protocorm-like body (PLB) of *Oncidium* hybrid orchid. The transgenic orchids show down-regulated level of *PSY* and geranyl synthase gene. They displayed semi-dwarf phenotype and brilliant green leaves. The microscopic anatomy revealed development-arrested plastids with rare grana. The total carotenoid content was decreased and the efficiency of the photosynthetic electron transport was declined. The chlorophyll level and the expression of chlorophyll biosynthetic genes, such as *OgGLUTR* and *OgCS* were dramatically reduced. HPLC analysis showed that the endogenous level of gibberellic acid and abscisic acid in the dwarf transformants are 4-fold lower than in wild type plants. In addition, chilling tolerance of the transgenic *Oncidium* plants was reduced. The data showed that down-regulation of *PSY* resulted in alterations of gene expression in enzymes involved in many metabolic pathways, such as carotenoid, gibberellic acid, abscisic acid and chlorophyll biosynthetic pathway as well as causes predominant defects in plant growth and development.

## Background

Carotenoids are the most widely distributed pigments and are essential components for all photosynthetic organisms (Fraser and Bramley 2004; Andrade-Souza et al. 2011). Carotenoid biosynthesis of higher plant occurs in plastids, and most carotenoids accumulate in chlorophyll–carotenoid–protein complexes in the thylakoid membranes. Therefore, carotenoid act as light-harvesting antenna pigments and optimize the efficacy of photosynthesis (Andrade-Souza et al. 2011). They supplement the light-capturing ability of chlorophyll by absorbing light in the range from 400 to 500 nm, a spectral region that is weakly absorbed by chlorophyll a (Telfer et al. 2008). Carotenoids also execute an important role in photoprotection in photosynthesis center. Furthermore, the protective effect of carotenoids in the blue region could be attributed to extra thylakoid carotenoids in ripening apples (Merzlyak et al. 2008). Photoprotection of carotenoids such as xanthophylls (zeaxanthin) is also shown by their role as antioxidants to scavenge singlet oxygen or superoxide anion radicals caused by light stress (Choudhury and Behera 2001). Finally, carotenoids are essential components in stabilization of the chloroplast apparatus (Havaux 1998).

In addition to the essential function in photoprotection and antioxidation, they act as precursors for the production of apocarotenoid hormones such as abscisic acid and strigolactones. The biosynthesis of carotenoid in plants has been well investigated (Bramley 2002; Fraser and Bramley 2004; Römer and Fraser 2005; Tanaka et al. 2008). The relevance of Arabidopsis as a model system for the study of carotenogenesis and how metabolic engineering approaches in this plant have taught important lessons for carotenoid biotechnology (Ruiz-Sola and Rodríguez-Concepción 2012). The first step of carotenogenesis is the dimerization of two molecules of geranylgeranyl pyrophosphate to phytoene by the phytoene synthase (PSY) (Lindgren et al. 2003). This step is the rate limiting step within the biosynthetic route and has long been considered a “bottleneck” in the pathway. Genes encoding phytoene synthase have been identified from numerous higher plants including tomato (Fraser et al. 2007), *Arabidopsis* (Maass et al. 2009), maize (Wong et al. 2004), citrus (Kato et al. 2004; Rodrigo et al. 2004), pepper (Tao et al. 2007), sunflower (Salvini et al. 2005), wheat (He et al. 2008) and maize (Li et al. 2008). Most plant species contain multiple functionally redundant copies of *PSY*, but different *PSY* genes appear to be differentially expressed and regulated.

Many efforts have been devoted to metabolic engineering of carotenoid biosynthesis in plants. Qin et al. (2007)) found that disruption of *phytoene desaturase* 3 (*PDS3*) in Arabidopsis resulted in an albino and dwarf phenotype by impairment of the chlorophyll, carotenoid and gibberellin (GA) biosynthesis. *PSY* overexpression has been studied in some plants. In tomato, overexpression of a fruit *PSY* caused dwarfism phenotype by competing GGPP from gibberellin pathway (Fray et al. 1995). In *Arabidopsis*, seed-specific overexpression of endogenous *PSY* resulted in delayed germination and increased levels of carotenoids, chlorophyll, and abscisic acid (Lindgren et al. 2003). Recently, overexpression of the maize *PSY* gene resulted in higher carotenoid levels in the rice endosperm, the so-called golden rice in which the pro-vitamin A content was increased (Paine et al. 2005). Although there are numerous cases of *PSY* overexpression in plants, silencing of this gene has not yet been reported.

Carotenoids also accumulate in flowers and fruits and are pivotal components for color patterns or nutrients, such as in the fruits of kiwi, banana, tomato, sunflower and several orchid species. The flowers of *Oncidium*“Gower Ramsey”, one of the most popular cultivars in Asian orchid industry, has a bright yellow ornamentation in the lip which is caused by violaxanthin, neoxanthin and lutein. This carotenoid mixture is concentrated in the chromoplasts of conical papillate cells of the adaxial epidermal layer (Hieber et al. 2006; Chiou et al. 2010). Moreover, the carotenoid composition determines the flower color in three cultivars of *Oncidium* orchids, *Gower Ramsey* (yellow), *Sunkist* (orange), and *White Jade* (white) (Chiou et al. 2010). Besides, the expression pattern of genes participating in carotenoid biosynthesis and metabolism showed also significant differences among the three cultivars, such as a high expression of *PSY* and *ZEP* in *Gower Ramsey,* while the carotenoid cleavage dioxygenase and 9-cis-epoxycarotenoid dioxygenase are highly expressed in *White Jade* (Chiou et al. 2010).

In a molecular breeding program for creating novel *Oncidium* cultivars with different floral color patterns, the RNA interference (RNAi) construct aiming at *PSY* was introduced into protocom-like bodies. Several transgenic orchids were obtained, displaying abnormal phenotypes in vegetative growth, such as yellowish-green leaves, smaller plant sizes and retarded growth. Our investigation demonstrated that the decrease of the carotenoid content was caused by down-regulation of the *phytoene synthase* gene in *OgPSY-RNAi* transgenic *Oncidium* plants. Phenotyping of the plants demonstrate that this impairment is caused by the growth-arrested chloroplast structure, the reduced chlorophyll level, and lower expression level of chlorophyll-biosynthetic genes. The potential physiological functions of carotenoids in plant growth and development are validated.

## Results

### Generation of transgenic *Oncidium*plants by RNA interference-based gene silencing of *phytoene synthase*

**Figure 1:**
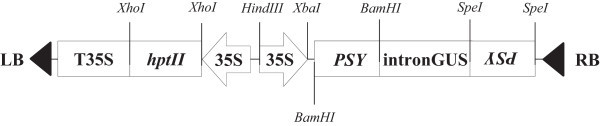
**Diagrammatic illustration of RNAi-based construct for silencing** ***phytoene synthase*** **in** ***Oncidium*** **hybrid.** *hptII*: *hygromycin phosphotransferase II*, Intron GUS: intron fragment of *β-glucuronidase*, LB/RB: left/right border of T-DNA, 35S: Cauliflower Mosaic virus 35S promoter, *PSY*: *phytoene Synthase.*

**Figure 2:**
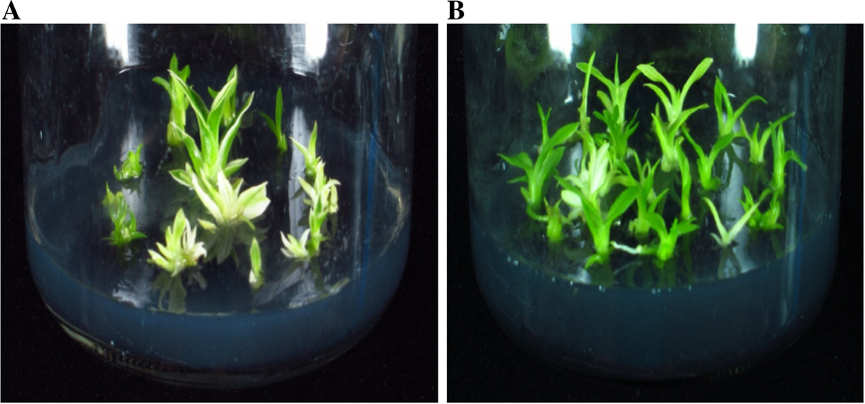
***Oncidium*** **shoots regenerating from protocorm-like bodies (PLBs). (A)** Shooting stage of transgenic *Oncidium* grown in medium with 10 mg L^-1^ hygromycin selection. **(B)** Wild type *Oncidium* grown in medium without hygromycin selection.

**Figure 3:**
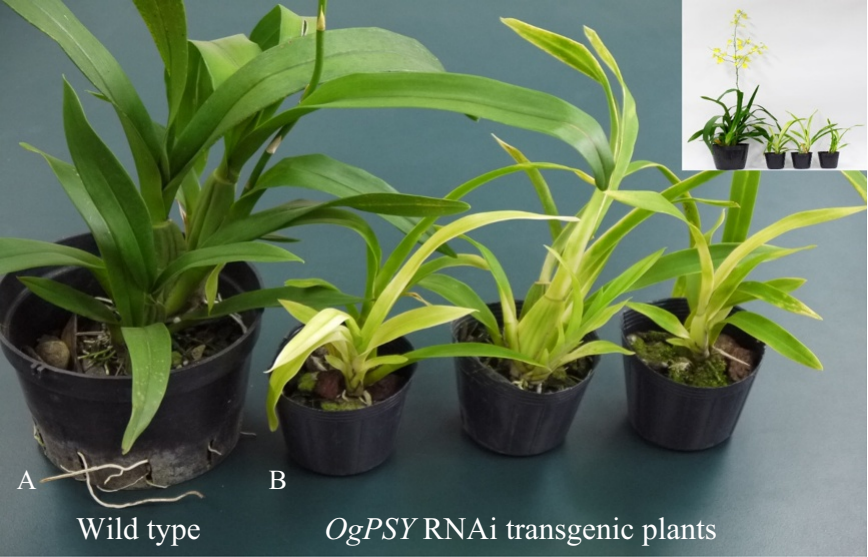
**The phenotype of Wild type and phytoene synthase RNAi transgenic** ***Oncidium*** **orchid. (A)** Wild type. **(B)** *OgPSY* RNAi transgenic plants. Plants are grown one year after removing from glass bottle. WT is blooming, and transgenic plants show growth retarded.

**Figure 4:**
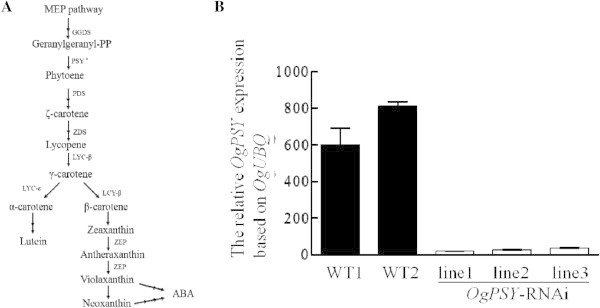
**Carotenoid biosynthesis pathway in** ***Oncidium*** **orchid and analysis of the expression level of** ***OgPSY*** **in transgenic** ***Oncidium*** **. (A)** The scheme of carotenoid biosynthesis pathway in *Oncidium* orchid. *Indicates the target gene of *PSY* for RNA interference-based gene silencing. **(B)** Relative transcript level of *OgPSY* gene in wild type and *OgPSY* RNAi transgenic *Oncidium* orchid. GGDS: *GGPP synthase*; PSY: *phytoene synthase*; PDS: *phytoene desaturase*; ZDS: *zeaxanthin desaturase*; LCY: *lycopene cyclase*. ZEP: *zeaxanthin epoxidase.* Gene transcript levels were measured by RT-qPCR. Transcript levels were calculated and normalized with respect to ubiquitin mRNA. The experiment was repeated three times.

The *OgPSY* RNAi construct driven by the 35S promoter was introduced into protocorm-like body (PLB) of *Oncidium* orchid by *Agrobacterium tumefaciens*-mediated transformation (Figure 1). After routine screening under three rounds of antibiotic (hygromycin) selection (Figure 2) for one year, six candidate transgenic lines were selected from hygromycin-containing MS-based medium and planted in pots. After an acclimated growth in the greenhouse for one year, all plants were assayed for the transgene expression. The transformation efficiency for the six transformants was confirmed for the expression of *hygromycin phosphotransferase* II (*hpt*II) by RT-PCR (data not shown). Moreover, all plants displayed down-regulated expression of *PSY* and a dwarf phenotype, when compared to wild type *Oncidium* plants (Figure 3). It confirms that the *PSY* gene is silenced by RNA-interference in the transgenic *Oncidium* orchids (Figure 4). Thus, three transgenic lines (line 1 ~ 3) were selected for further molecular and physiological analyses.

### *PSY-RNAi*in transgenic *Oncidium*alters the expression pattern of biosynthetic pathway and leads to decreased accumulation of carotenoid contents

**Figure 5:**
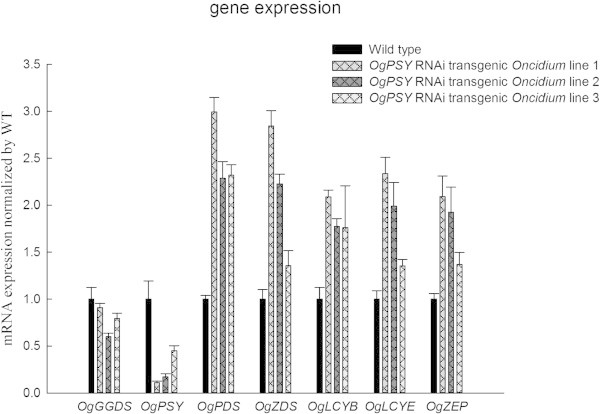
**Relative transcript levels of carotenoid biosynthesis genes in transgenic** ***Oncidium*** **orchids.** Transcript levels of the carotenoid biosynthesis genes. GGDS: *GGPP synthase*; PSY: *phytoene synthase*; PDS: *phytoene desaturase*; ZDS: *zeaxanthin desaturase*; LCY: *lycopene cyclase*. ZEP: *zeaxanthin epoxidase.* Gene transcript levels were measured by RT-qPCR. Transcript levels were calculated and normalized with respect to ubiquitin mRNA. The experiment was repeated three times.

**Figure 6:**
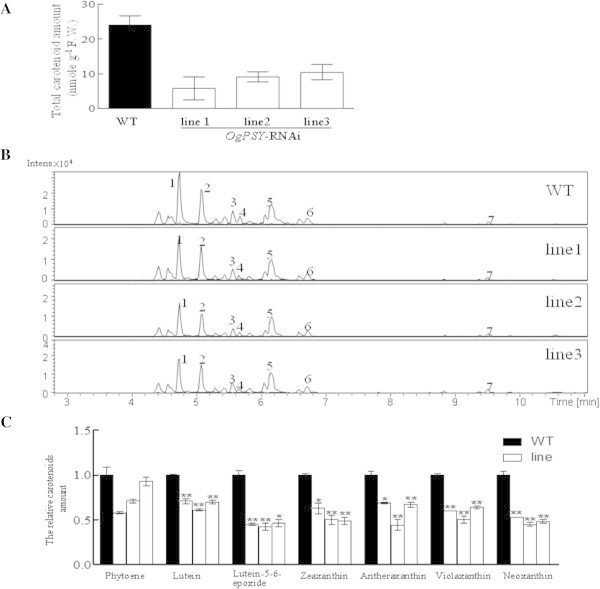
**The carotenoid amount, LC-Ms profiles of carotenoid fingerprints and component quantitation in transgenic** ***Oncidium*** **plants. (A)** Total carotenoid amount; **(B)** LC-Ms profiles of carotenoid components. No.1-7 represent neoxanthin, violaxanthin, antheraxanthin, lutein-5-6-epoxide, lutein, zeaxanthin and phytoene, respectively. **(C)** Quantitation of carotenoid component. The experiment was repeated three times.

The expression levels of *phytoene synthase* and the other step genes involved in the carotenoid biosynthetic pathway were monitored in overall. As shown in Figure 5, *OgPSY* was predominantly reduced in all three transgenic orchid plants, compared to wild type. The expression of *GGDS* gene, which is responsible for the synthesis of geranyl geranyl pyrophosphate (GGPP) was also reduced slightly. However, all the downstream genes, such as *phytoene desaturase* (*OgPDS*), *lycopene cyclaseB, E* (*OgLCYB* and *OgLCYE*), *zeaxanthin desaturase* (*OgZDS*) and zeaxanthin *epoxidase* (*OgZEP*) were dramatically upregulated, compared to wild type. The carotenoid contents in three transgenic lines were subject to assay. Analysis by LC-ESI-MS system showed that the total carotenoid content, neoxanthin, violaxanthin, antheraxanthin, lutein-5-6-epoxide, lutein, zeaxanthin and phytoene were significantly reduced (Figure 6). Some components, such as neoxanthin, lutein-5-6-epoxide and zeaxanthin were reduced to 50% level.

### The chloroplast apparatus and chlorophyll level are impaired in *PSY RNAi*-transgenic *Oncidium*orchids

**Figure 7:**
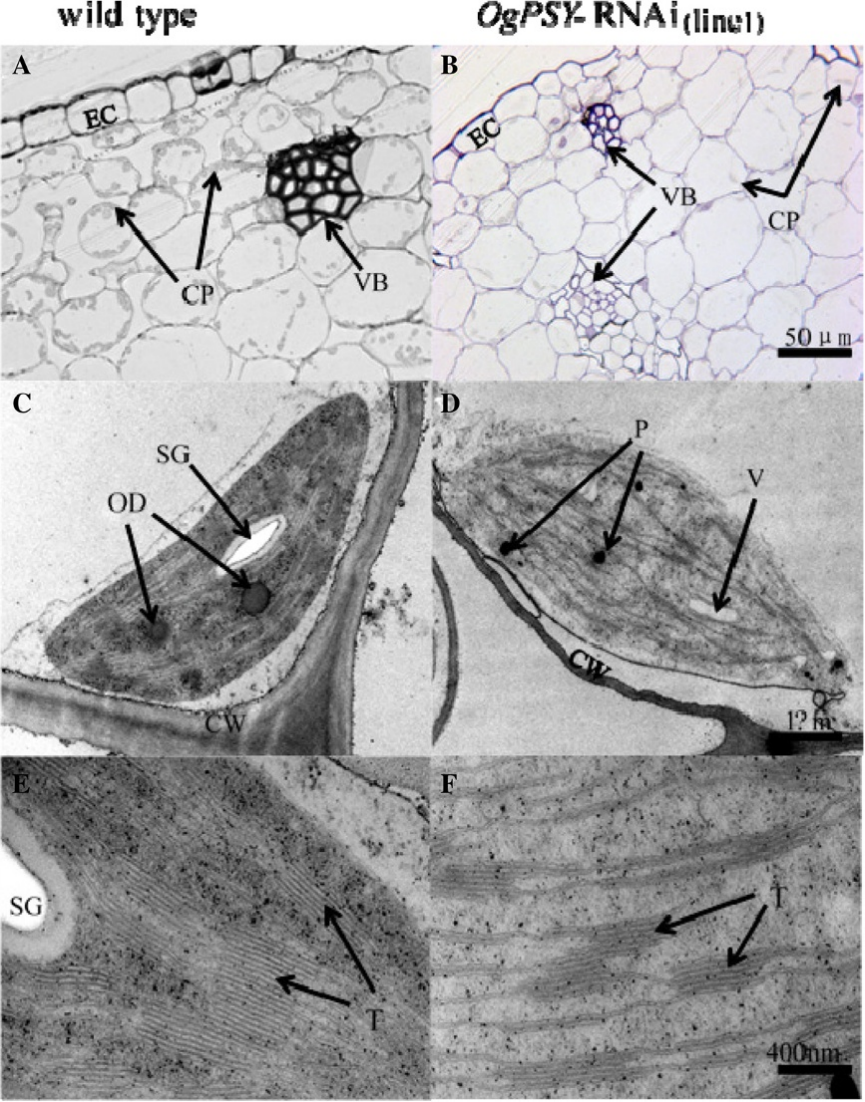
**The distribution and structure of chloroplasts in the leaf mesophyll cells of wild type and** ***OgPSY*** **-RNAi transgenic** ***Oncidium*** **.** **(A,** **C and** **E)**, Wild-type *Oncidium*; **(B,** **D and** **F)**, *OgPSY*-RNAi transgenic *Oncidium*. EC: epidermal cell CP: chloroplast; VB: vascular bundle; SG: starch grain; OD:oil droplet; P: Plastoglobuli; CW : cell wall;V: vacuolization; T: thylakoid.

The *PSY RNAi*-transgenic orchids exhibit an abnormal phenotype such as yellow-greenish leaves and dwarfism, when compared to wild type plants (Figure 3). Therefore, the anatomical structures of the plastids were analyzed by microscopy. As shown in Figure 7A and B, the number of plastids in leaves of the transgenic orchids were decreased, compared to wild type plants. The chloroplasts in wild type orchid leaves contained the well-known dense grana structure, oil bodies and starch grains, whereas plastids in the transgenic orchids were arrested at a proplastid-like stage. They contain barely any grana, no starch grains and oil droplets (Figure 7C, D). For some plastids of the transgenic plants multi-plastoglobuli, blurry membrane edges, and vacuolization phenomena inside the organelle was observed. Most obviously, the plastids of the transgenic lines had very little thylakoid lamellar structures and very sparse grana stacks, compared to the wild type (Figure 7E, F). The thickness of grana lamellas of wild-type plastids was approximately 18.02 nm, whereas that of the transgenic lines was 20.24 nm. It indicates that grana lamellae cannot be compactly organized in the transgenic orchids.

**Figure 8:**
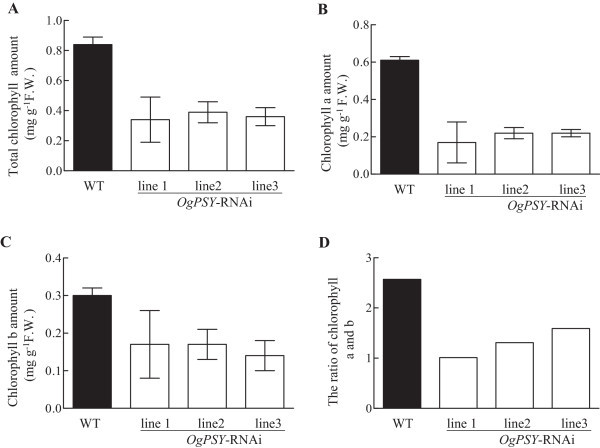
**The amount of total chlorophyll, chlorophyll a, chlorophyll b and the chlorophyll a/b ratios in wild-type and** ***OgPSY-RNAi*** **transgenic** ***Oncidium*** **. (A)** Total chlorophyll; **(B)** Chlorophyll a; **(C)** Chlorophyll b and **(D)** The chlorophyll a/b ratios in wild-type and *OgPSY-RNAi* transgenic *Oncidium*. The experiment was repeated three times.

Furthermore, the total chlorophyll a and b content was significantly reduced to approximately1/3 of wild type in the three transgenic lines (Figure 8A-C). The decrease of the chlorophyll a/b ratio further demonstrates that decrease of chlorophyll a biosynthesis is more impaired than chlorophyll b biosynthesis. The chlorophyll a/b ratio is an indicator for the efficiency of light adaptation/acclimation of the photosynthetic apparatus. Chlorophyll b is an antenna component, whereas chlorophyll a is present in the reaction center of photosystem I and II and the antenna (Telfer et al. 2008). The decrease of chlorophyll a suggests that the reaction centers of PSI and PSII are impaired. Since the chlorophyll a content is higher in the light-harvesting LHC-I complex of PSI, the PSI antenna may be more impaired than the PSII antenna.

### The decreased carotenoid level resulted in reduced expression levels of chlorophyll biosynthetic genes

**Figure 9:**
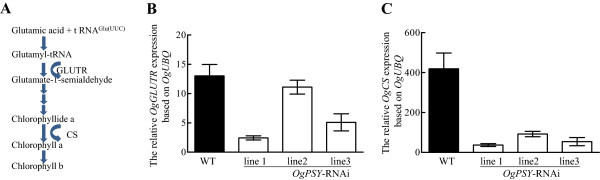
**Chlorophyll biosynthesis pathway in plant and the expression level of** ***OgGLUTR*** **and** ***OgCS*** **in wild-type and** ***OgPSY-RNAi*** **transgenic** ***Oncidium*** **. (A)** The scheme of chlorophyll biosynthesis pathway in plant. **(B) (C)** Transcript levels of *OgGLUTR* and *OgCS* in wild-type and transgenic plants. Gene transcript levels were measured by RT-qPCR. Transcript levels were calculated and normalized with respect to ubiquitin mRNA. The experiment was repeated three times.

Besides reduced carotenoid content, the *PSY RNAi*-transgenic *Oncidium* orchids contain also less chlorophyll levels. The expression of the two chlorophyll biosynthesis-related genes *OgGLUTR* and *OgCS*, which are the first and the last rate-limiting genes in chlorophyll biosynthesis (Figure 9A), were assayed by qPCR. The expression levels of *OgGLUTR* and *OgCS* are severely reduced in the three transgenic lines (Figure 9B, C), and the effect was stronger for *OgCS*. The data demonstrate that the expression of chlorophyll biosynthesis genes is repressed in the nucleus, suggesting a cross-talk between the two pigment synthesis pathways.

### *PSY RNAi*-transgenic *Oncidium*orchids are impaired in photosynthetic efficiency in PSII and PSI activity

PSII and PSI activity were measured by Dual-PAM-100 fluorometer. As shown in Table 1, both Fv/Fm and Y (II) values of the three transgenic lines were less than those of wild type plants, whereas the Y (NO) and Y (NPQ) values were higher than the wild type plants. A high Y (NO) value indicates that the photochemical energy conversion and the protective regulatory mechanisms are inefficient in the transgenic lines. A high Y (NPQ) value indicates that the photon flux density is excessive and that the plants have retained the physiological means to protect themselves by regulation, i.e. the dissipation of excessive excitation energy into heat. Therefore, the data indicate that the photon flux density was excessive for the three transgenic lines, and that they would be photo damaged upon further irradiation.

**Table 1:** **The photosynthetic properties of** ***OgPSY-RNAi*** **transgenic** ***Oncidium*** **and wild type**

	Fv/Fm	Y (II)	Y (NO)	Y (NPQ)	Pm	Y (I)	Y (ND)	Y (NA)
**WT**								
CK1	0.80	0.76	0.21	0.03	0.82	0.90	0	0.11
CK2	0.82	0.78	0.19	0.03	0.80	0.83	0	0.17
***OgPSY-RNAi Oncidium***								
line 1	0.78	0.73	0.23	0.04	0.24	0.74	0.03	0.23
line 2	0.80	0.75	0.21	0.04	0.42	0.78	0	0.22
line 3	0.77	0.69	0.25	0.06	0.19	0.75	0.08	0.17

Furthermore, the Pm and Y (I) values for the three transgenic lines were lower than those for the two wild type controls, and the effect is more dramatic for the Pm value. Pm represents the maximal change of the P700 signal upon quantitative transformation of P700 from the fully reduced to the fully oxidized state (Klughammer and Schreiber 1994). Y (I) is the photochemical quantum yield of PSI. The values of Y (ND) and Y (NA) of the three transgenic lines were higher than those of the two wild type plants. Y (ND) is a measure of the donor side limitation which is enhanced by a trans-thylakoid proton gradient and damage at the level of PSII. Y (NA) is a measure of acceptor side limitation, which is enhanced by damage at the level of CO_2_ fixation. Thus, not only PSII and PSI but also CO_2_ fixation is impaired in the three transgenic lines. Compared to the WT, the Pm and Y (I) values of PSI in three transgenic lines declined by 64.8% and 12.7% respectively, whereas the Fv/Fm and Y (II) values of PSII declined only by 3.5% and 5.7%, respectively (data not shown). This indicates that PSI is more severely damaged than PSII in the *PSY-*RNAi transgenic *Oncidium* orchids. This is consistent with the reduced content of chlorophyll in PSI (Figure 8).

### The transgenic plants do not accumulate more H_2_O_2_

**Figure 10:**
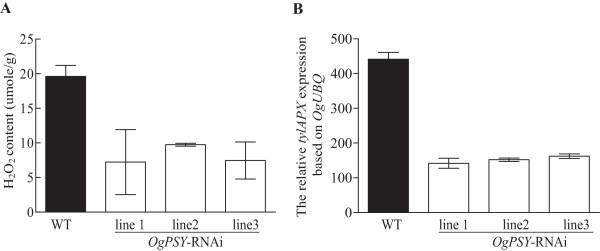
**H**_**2**_ **O**_**2**_ **levels and relative transcript level of** ***tylAPX*** **in wild-type and** ***OgPSY*** **-RNAi transgenic** ***Oncidium*** **. (A)** H_2_O_2_ levels; **(B)** Transcript levels of *tylAPX* in wild type and *OgPSY* RNAi transgenic plants. Gene transcript levels were measured by RT-qPCR. Transcript levels were calculated and normalized with respect to ubiquitin mRNA. The experiment was repeated three times.

H_2_O_2_ accumulation , one of the reactive oxygen species (ROS) generated in the plastids (del Rio et al. 2006; Navrot et al. 2007), is dramatically enhanced in response to environmental stress conditions, which results in an excessive demand of H_2_O_2_-accumulationg cells for an cellular efficient antioxidant machinery (Fahnenstich et al. 2008). Ascorbate (AsA) is a reductant of H_2_O_2_ in chloroplasts. Ascorbate peroxidase (APX) utilizes AsA as its specific electron donor to reduce H_2_O_2_ to water with the concomitant generation of monodehydroascorbate (MDAsA) (Shigeoka et al. 2002). The tylAPX (thylakoid form) is a plastic H_2_O_2_-scavenging enzyme to protect chloroplast from ROS damage. The H_2_O_2_ levels and the expression of the *tylAPX* gene in leaves were measured. The three transgenic lines had a low H_2_O_2_ content (Figure 10A). Also, the expression level of *tylAPX* was lower than in the wild type (Figure 10B). Therefore, the results demonstrate that the decreased photosynthetic efficiency and plastid impairment in the transgenic orchids are not caused by the H_2_O_2_ accumulation, but due to the impaired carotenoid biosynthesis.

### Silenced expression of *PSY*in transgenic *Oncidium*leads to the decrease of endogenous ABA and GA levels

**Figure 11:**
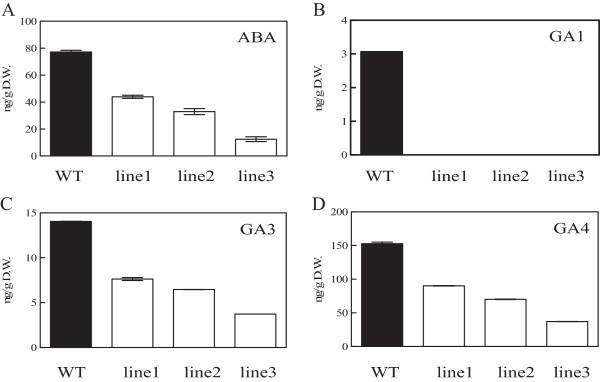
**The analysis of ABA and GA levels in wild type and** ***OgPSY*** **-RNAi transgenic** ***Oncidium.*** The **(A)** ABA; **(B)** GA1; **(C)** GA3 and **(D)** GA4 levels in wild type and *OgPSY* RNAi transgenic plants. The experiment was repeated three times.

We noticed that the transgenic *Oncidium* orchids are very susceptible to a 12°C chilling treatment (data not shown). ABA, a stress-inducible isoprenoid plant hormone, is one of the final products in the carotenoid biosynthesis pathway. Similarly, GA is synthesized in plastids and participates in stress responses. Therefore, the endogenous ABA and GA levels were monitored. Both phytohormone levels were reduced in the transgenic *Oncidium* orchids (Figure 11). GA_1_, GA_3_ and GA_4_ were reduced in transgenic orchids, whereas GA_1_ was almost deficient in the three transgenic orchids (Figure 11B-D). The data show that carotenoid shortage also affects the biosynthesis of GA and ABA.

## Discussion

In this study, the 35S::*PSY*-RNAi construct was delivered to *Oncidium* GR hybrid orchid by *Agrobacterium tumefaciens*-mediated infection. The transformed *Oncidium* orchid shows a constitutive down-regulated expression level of *PSY* (Figure 4). The transgenic orchids show a very strong phenotype, such as dwarfism, bleached leaves (Figure 2), retarded growth and unbolting (Figure 3). The carotenoid and chlorophyll contents are dramatically decreased in the transgenic lines (Figures 6 and 8). They also have severely reduced GA and ABA levels (Figure 11). Additional physiological defects, such as low photosynthesis efficiency (Table 1) and low chilling tolerance were also observed (data not shown). Although the transgenic *Oncidium* orchids did not show the expected floral color modification, the results demonstrate an important function of the phytoene synthase, which is not restricted to carotenoid biosynthesis alone.

The qPCR data demonstrated that the expression level of *phytoene synthase* is dramatically reduced, but expression levels of the downstream genes, such as *OgPDS*, *OgZDS*, *OgLCYE*, *OgLCYB* and *OgZEP* are relatively increased in *PSY*-RNAi *Oncidium* where contents of carotenoid were decreased (Figure 5). This feedback regulation between carotenoid level and its biosynthetic genes have been described in plants. Diretto et al. (2010) demonstrated that the high levels of carotenoids and β-carotene in “Golden” potato tubers which were engineered by three bacterial genes forβ-carotene synthesis seem to trigger a repression of endogenous *PSY1* and an induction of β-xanthophyll synthases and lycopeneϵ-cyclase. In Peach, the expression level of *PSY*, *ZDS* and *CHY-B* genes at final ripening stages in yellow-fleshed cultivar ‘Redhaven’ (rich carotenoid content) were lower than white-fleshed bud sport mutant cultivar ‘Redhaven Bianca’ (low carotenoid content ) (Brandi et al. 2011).

The LC-MS analysis showed that phytoene, lycopene, β-carotene and other carotenoid compounds were reduced. This is caused by the down-regulation of the *phytoene synthase* gene in transformed orchids. ABA synthesis is also reduced as a downstream effect of the inhibition of the carotenoid metabolic pathway. Chlorophyll contents and GA levels were reduced in the mutants. Chlorophyll biosynthesis and GA biosynthesis are branches of the carotenoid biosynthetic pathway. Both are synthesized from the common substrate geranylgeranyl pyrophosphate (GGPP). Theoretically, reduction of the amount of phytoene and of phytoene synthase activity may cause a large accumulation of GGPP, which should result in a massive biosynthesis of chlorophyll and GA. The reverse results indicated that there might be a feedback mechanism when the carotenoid biosynthetic pathway is blocked at the step of phytoene synthase. Interestingly, the result is similar to *phytoene desaturase* studies in *Arabidopsis*, where *pds* insertional knock-out mutants showed dwarfism and a low chlorophyll/carotenoid content (Qin et al. 2007).

Carotenoid biosynthesis is an important metabolic pathway in plants. The biosynthesis of phytohormones (GA and ABA), chlorophyll, vitamins and other essential pigments for plastids are closely related to this pathway (Meier et al. 2011). Carotenoids and chlorophyll play an important structural role in the assembly of light-harvesting complexes. The major pigment-binding proteins in plant chloroplast membranes are light-harvesting complex II (LHC-II). The proteins are estimated to represent about half of the total proteins in the thylakoid membrane (Telfer et al. 2008). The complex binds chlorophyll a, chlorophyll b and carotenoids (Pilkington et al. 2012; Kusaba et al. 2007). Because carotenoids together with chlorophylls are components of these membrane associated pigment-protein complexes, assembly of the light-harvesting complexes is dependent on the presence of chlorophylls and carotenoids (Sagar and Briggs 1990). Recently, DELLA proteins were known to coordinate the biosynthesis of chlorophyll and carotenoids through the regulation of PIF activity in *Arabidopsis* (Cheminant et al. 2011). Inhibition of PSY-biosynthesis blocks therefore thylakoid membrane assembly.

The transgenic orchids have photo-bleached leaves (Figures 2 and 3) and contained proplastid-type plastids (Figure 7). There are two possible interpretations. One is that carotenoids are membrane stabilizers in chloroplasts (Havaux 1998). Carotenoids of the xanthophyll family and some other terpenoids, such as isoprene or α-tocopherol, stabilize and photoprotect the lipid phase of the thylakoid membranes. When plants are exposed to potentially harmful environmental conditions such as strong light intensity,the xanthophyll violaxanthin and the products of its enzymatic de-epoxidation, antheraxanthin and zeaxanthin, partitions in light-harvesting complex functions and lipid organization of the thylakoid membranes. The resulting interaction of the xanthophyll molecules with the membrane lipids is responsible for a decrease in membrane fluidity, an increase in membrane thermo stability and a lowered susceptibility to lipid peroxidation (Havaux 1998). The decrease of the carotenoid content in our studies causes an unusual thylakoid membrane assembly, or causes lipid phase changes of the membrane structure, which lead to the described phenotypes (Figure 7D, F). Alternatively, repression of chlorophyll biosynthesis (Figure 9) and the reduced chlorophyll level in the mutant (Figure 8) cause the development arrest of the plastids (Figure 7D, F), because of the carotenoids are limiting a proper assembly of the thylakoid membrane structures.

The transgenic orchids displayed a severe dwarf phenotype, such as retarded growth, short leaves and absence of bolting (Figure 3). This outcome is possible caused by GA shortage and low photosynthesis efficiency. Assays of GA_1_, GA_3_ and GA_4_ showed that these hormone levels are extremely low in transgenic orchids, the GA_1_ amount in all transgenic lines compared to WT was almost not detected; whereas the amounts of GA_3_ and GA_4_ in all transgenic lines exhibited 30 ~ 50% compared to WT (Figure 11B-D). It suggests that the growth potential is distinctly impeded. In addition, the photosynthetic efficiency of the photosynthetic apparatus is severely declined in the transgenic orchids (Table 1). The decrease of photosynthesis products also might cause energy deficiency in plants and be responsible for the observed phenotype. The carotenoid deficiency in transgenic *Oncidium* orchids also leads to reduced ABA levels, which in turn causes the low chilling stress tolerance (data not shown).

It is known that the expression of nuclear-encoded photosynthetic genes is inhibited through signals from plastids (Taylor 1989). Recently, several studies described the effects of carotenoid deficiency on the expression of photosynthetic genes. Norflurazon-treated *Arabidopsis* seedlings showed that expression of 182 genes, including photosynthetic genes were repressed more than 3-fold (Sagar and Briggs 1990). In addition, a *pds*3 mutant with severe carotenoid deficiency showed predominant repression of photosynthetic genes (Qin et al. 2007). Moreno et al. (2013) also demonstrated that a pronounced reduction in storage root thickness and color of carrots was obtained in *DcLcyb1* transgenic silenced lines.

It was reported that *PSY* overexpression caused plants a reduced GA level, due to the increasing utilization of GGPP for carotenoid biosynthesis, and leading to a dwarf phenotype (Fang et al. 2008; Fray et al. 1995). However, the *PSY*-RNAi transgenic orchids in our work also displayed the similar outcome (Figure 11B-D). It suggests that plant carotenoidgenesis pathway is well fine-tuned to regulate. Taken together, we showed that down-regulation of *phytoene synthase* caused carotenoid deficiency and severely reduced GA and ABA levels, as well as a severe negative effects on plant’s performance. In accordance with previous reports, the mechanisms of feedback gene regulation and the responsible signaling pathways are still attracting much attention.

## Conclusions

To the best of our knowledge, our work is the first report that using RNAi approach to break down carotenoidgenesis in *Oncidium* hybrid orchid. The data indicate that the down-regulation of *PSY* resulted in gene expression changes involved in many metabolic pathways, such as carotenoid, gibberellic acid, abscisic acid and chlorophyll biosynthetic pathway, in addition to phenotypic defect.

## Materials and methods

### Plant materials

*Oncidium* Gower Ramsey hybrid was provided by Chinese Development Association for *Oncidium* Production and Marketing (CDAOPM) at Chiayi, Taiwan, and the transgenic plant lines were obtained by transforming with the *OgPSY-RNAi* vector. Orchid seedlings were grown in the greenhouse at a temperature range of 20-28°C with 14 h photoperiod.

### Generation of transgenic plant and selection of transgenic plants

The protocom-like bodies (PLBs) of *Oncidium* Gower Ramsey were generated and maintained onto G10 medium containing MS salts, 1 g/L tryptone, 20 g/L sucrose, 1 g/L charcoal, 65 g/L potato tubers, and 3 g/L phytagel at pH 5.4. The transformation protocol was carried out according to Liau et al. (2003). The binary vector pCAMBIA 1390 (Figure 1) containing the *OgPSY*-RNAi construct driven by the cauliflower mosaic virus 35S promoter (P35S) introduced into PLB by *A. tumefaciens* EHA105-mediated infection. The transformed seedlings were screened after several rounds of selection on hygomycin-containing medium.

### Real-time quantitative PCR measurement for gene expression level

The primers for real-time quantitative PCR were designed by using the Primer Express Software v3.0 (Applied Biosystems, St. Louis, MO); gene-specific primers for *OgGGDS, OgPSY, OgPDS, OgZDS, OgLCYB, OgLCYE, OgZEP*, *OgCS, OgGLUTR, OgtylAPX* and *UBQ* are given in Table 2. Total RNA was extracted and cDNA was synthesized following the RevertAidTM first strand cDNA systhesis kit protocol (Fermentas). Real-time RT-PCR reaction was performed following the instruction of the KAPA SYBR FAST qPCR Kit (Kapa Biosystems) by using the ABI 7500 Fast Real-Time PCR system (Applied Biosystems). All reactions were repeated three times.

**Table 2:** **List of primers for PCR systems**

Gene	Primer name	Sequence
*OgGGDS*	Fp	5′ -GCGAGCGATAAGACGACGTAT-3′
	Rp	5′ -AAGCAATATAATTCGCCAGACAAAG-3′
*OgPSY*	Fp	5′ -CTCCAATTAACTGTAGCGCATCTTC-3′
	Rp	5′ -CATTCTTCGCGTTCCATAACC-3′
*OgPDS*	Fp	5′ -CTTCTTGGGTCTATTTCTACTGGGTTT -3′
	Rp	5′ -ACACCCCATGTAATCACTGCTTCT -3′
*OgZDS*	Fp	5′ -AATCGGCCATGGCTTCTG -3′
	Rp	5′ -GCTCGGGCGGAAATAACC -3′
*OgLCYB*	Fp	5′ -CGGAGGTGTCGGCTAGGGTTTGG -3′
	Rp	5′ -CCAACCTACACCCAGCCGAAGCG -3′
*OgLCYE*	Fp	5′ -TCCACACTTTCTTCGGCGGGTCTTATA -3′
	Rp	5′ -CTCCACATTAAGTTCACAAGGTAAGGTATTCCC -3′
*OgZEP*	Fp	5′ -CCTCCAAAGGAATCGGGTTT -3′
	Rp	5′ -CCGGCGATGAGAATACGAAA -3′
*OgCS*	Fp	5′ -GCTTTTTTCCTGGTGGTTAGTGA-3′
	Rp	5′ -CGTGGTGAGTCGAGGTGAAAC-3′
*OgGLUTR*	Fp	5′ -CAGCAAGGCAAGATCGGAAA-3′
	Rp	5′ -ACCAGACCTCCTCAGAAACATCAA-3′
*OgtylAPX*	Fp	5′ -TCTTCTTGTTTTACCTACTGATGCCATA-3′
	Rp	5′ -CTGAGCGTACTTCTCTGCATAGATCTT-3′
*UBQ*	Fp	5′ -GCATGCAAGCTTGGCGTAA -3′
	Rp	5′ -TGAGCGGATAACAATTTCACACA -3′

### Measurement of the chlorophyll and carotenoid content

The extraction and analysis of pigments were carried out following the method by Schubert et al. (2006). In brief, the pigments were extracted with appropriate volume of dimethylformamide (DMF) for 24 h at 4°C in dark after the leaf tissues were ground in liquid nitrogen. The ground tissues were immediately centrifuged twice to separate debris. After the supernatant was removed, the carotenoid pigments were quantitatively measured by a spectrophotometer (TECAN Infinite® M200 PRO) at A_450_ following the method by Moran and Porath (1980). Chlorophyll a, b and total chlorophyll were quantitatively measured at A_663_ and A_645_, before calculation of the concentration according to Arnon (1949).

### Measurement of carotenoids

The extraction was carried out by adding 40 μL of 100% DMF to 1 mg of the dry plant powder with vortexing for 30 min. After centrifugation at 16,000 × g for 10 min, the supernatant was collected for LC-ESI-MS analysis. The LC-ESI-MS system consisted of an ultra-performance liquid chromatography (UPLC) system (Ultimate 3000 RSLC, Dionex) and an electrospray ionization (ESI) source of quadrupole time-of-flight (TOF) mass spectrometer (maxis HUR-QToF system, Bruker Daltonics). The autosampler was set at 4°C. Separation was performed with reversed-phase liquid chromatography (RPLC) on a BEH C8 column (2.1 × 100 mm, Walters). The elution started from 50% mobile phase A (0.1% formic acid in deionized water) and 50% mobile phase B (0.1% formic acid in ACN), raised to 60% B in 2 min, further increased to 75% after an additional 1 min, and then raised to 100% B in 4.5 min, held at 100% B for 2.5 min, and then lowered to 50% B during the final 1 min. The column was equilibrated by pumping 50% B for 4 min. The flow rate was set 0.4 mL/min with an injection volume of 2 μL. LC-ESI-MS chromatogram was acquired in positive ion mode under following conditions: capillary voltage of 4500 V, dry temperature at 190°C, dry gas flow maintained at 8 L/min, nebulizer gas at 1.4 bar, and acquisition range of m/z 100–1000.

### Chloroplast structure under transmission electron microscopy (TEM)

Leaves were fixed in 2.5% glutaraldehyde in 0.1 M sodium phosphate buffer (pH 7.2) at 4°C overnight. After 3 washes 20 min each, tissues were post fixed in 1% OsO_4_ in the same buffer for 4 h at room temperature, and then rinsed again three times in fresh buffer (20 min each). The leaves were dehydrated in an acetone series, embedded in Spurr’s resin, and sectioned with a Leica ultracut E ultramicrotome. Semi-thin sections (1 μm) for light microscopy were placed on slides and stained with 0.1% toluidine blue for 1 min at 60°C on a hot plate. Thin sections on grids were stained with uranyl acetate and lead citrate and examined with a Philips CM 100 transmission electron microscope (TEM) at 80 KV.

### Measurement of chlorophyll Photosystem II (PSII, P680) and Photosystem I (PSI, P700)

The chlorophyll fluorescence of PSII and the redox state of P700, an indicator of PSI activity, were measured *in vivo* concomitantly at room temperature using a Dual-PAM-100 fluorometer (Walz) connected to a PC with WinControl software. After dark adaptation of the leaves for 20 min, experiments were carried out by using the automated induction and recovery curve routine in the Dual-PAM software, with repetitive application of saturation pulses for the assessment of fluorescence and P700 parameters from which the quantum yields of PSI and PSII were calculated by the software. The fluorescence parameters were calculated as follows: Fv/Fm = (Fm-Fo)/Fm, Y (II) = (Fm'-F)/Fm' (Genty et al. 1989), Y (NO) = 1/ (NPQ + 1 + qL (Fm/Fo-1) (Kramer et al. 2004), Y (NPQ) = 1-Y (II) -1/(NPQ + 1 + qL (Fm/Fo-1) (Kornyeyev and Hendrickson 2007; Kramer et al. 2004), Y (I) = 1- Y (ND) -Y (NA), Y (ND) = 1-P700 red, Y (NA) = (Pm-Pm') /Pm.

### Measurement of H_2_O_2_ content

The concentration of H_2_O_2_ in leaves was measured following the method described by Jana and Choudhuri (1982). All experimental steps were performed at 4°C. 0.125 g leaf tissue was ground to a fine powder in liquid nitrogen and further homogenized with 450 μL Solution I (10 mM Na-P buffer, pH 6.5, 10 mM 3-amino-1.2.4-triazole). The supernatant from a 25 min centrifugation at 13,000 g under 4°C was transferred to a new eppendorf tube and the extraction was repeated. To 600 μL of the supernatant 200 μL Solution II (0.1% titanium oxysulfate and 0.1% titanium sulfate in 20% H_2_SO_4_) was added and the solution was mixed well. . Finally, 200 μL supernatant was dispensed to 96 well plates and the absorption was measured at OD_410_ (TECAN Infinite® M200 PRO). The H_2_O_2_ content was calculated with a standard curve.

### Determination of abscisic acid and gibberellic acid levels in *Oncidium*orchid leaves

Endogenous ABA and GA were analyzed by gas chromatography–mass spectrometry-selected ion monitoring (GC-MS-SIM). The fresh leaves were lyophilized, weighted and stored at -20°C. The measurement and analyses were performed according to Chen et al. (2010), and each sample was repeated twice.

### Statistical analysis

All data are presented as a mean and standard deviation. Data were subjected to a one way analysis of variance (ANOVA) using SAS v.9.2 (SAS Institute, Cary, NC, USA). P < 0.05 was considered statistically significant.
